# *In Vitro* Antimetastatic Effect of Phosphatidylinositol 3-Kinase Inhibitor ZSTK474 on Prostate Cancer PC3 Cells

**DOI:** 10.3390/ijms140713577

**Published:** 2013-06-28

**Authors:** Wennan Zhao, Wenzhi Guo, Qianxiang Zhou, Sheng-Nan Ma, Ran Wang, Yuling Qiu, Meihua Jin, Hong-Quan Duan, Dexin Kong

**Affiliations:** 1 Tianjin Key Laboratory on Technologies Enabling Development of Clinical Therapeutics and Diagnostics, School of Pharmaceutical Sciences, Tianjin 300070, China; E-Mails: zhaowennan@tijmu.edu.cn (W.Z.); shiliuhao@hotmail.com (W.G.); zqx9260@163.com (Q.Z.); jane6386812@126.com (S.-N.M.); wangran_2010@hotmail.com (R.W.); potato252525@hotmail.com (Y.Q.); jinmeihua@tijmu.edu.cn (M.J.); duanhq@tijmu.edu.cn (H.-Q.D.); 2 Research Center of Basic Medical Sciences, Tianjin Medical University, Tianjin 300070, China; 3 Division of Molecular Pharmacology, Cancer Chemotherapy Center, Japanese Foundation for Cancer Research, 3-8-31, Ariake, Koto-ku, Tokyo 135-8550, Japan

**Keywords:** phosphatidylinositol 3-kinase inhibitor, antimetastasis, ZSTK474, PC3, Girdin, VEGF

## Abstract

Tumor metastasis is the main cause of lethality of prostate cancer, because conventional therapies like surgery and hormone treatment rarely work at this stage. Tumor cell migration, invasion and adhesion are necessary processes for metastasis. By providing nutrition and an escape route from the primary site, angiogenesis is also required for tumor metastasis. Phosphatidylinositol 3-kinases (PI3Ks) are well known to play important roles in tumorigenesis as well as metastasis. ZSTK474 is a specific PI3K inhibitor developed for solid tumor therapy. In the present report, antimetastatic activities of ZSTK474 were investigated *in vitro* by determining the effects on the main metastatic processes. ZSTK474 exhibited inhibitory effects on migration, invasion and adhesive ability of prostate cancer PC3 cells. Furthermore, ZSTK474 inhibited phosphorylation of Akt substrate-Girdin, and the secretion of matrix metalloproteinase (MMP), both of which were reported to be closely involved in migration and invasion. On the other hand, ZSTK474 inhibited the expression of HIF-1α and the secretion of vascular endothelial growth factor (VEGF), suggesting its potential antiangiogenic activity on PC3 cells. Moreover, we demonstrated the antiangiogenesis by determining the effect of ZSTK474-reduced VEGF on tube formation of human umbilical vein endothelial cells (HUVECs). In conclusion, ZSTK474 was demonstrated to have potential *in vitro* antimetastatic effects on PC3 cells via dual mechanisms: inhibition of metastatic processes including cell migration, invasion and adhesion, and antiangiogenesis via blockade of VEGF secretion.

## 1. Introduction

Prostate cancer is currently the most prevalent tumor in men and the second leading cause of cancer-related deaths in the western world. Since prostate cancer is a disease of the aging male, with aged population increasing in developed countries as well as some developing countries like China, incidence of prostate cancer would be even higher in the world. Prostate cancer is often diagnosed in the advanced stage because development of this disease is asymptomatic. In the advanced stage, tumor metastasis is often observed, which makes conventional treatments such as surgery and hormone therapy rarely work. Therefore, patients die of prostate cancer because of metastasis.

Metastasis arises following the spread of cancer from a primary site and the formation of new tumors in distant organs. A series of processes are involved in metastasis: angiogenesis for supplying nutrition and escape route; escape of primary tumor cells from the original tumor site; intravasation; survival in the circulation system; extravasation into a distant organ; and proliferation there [1]. Therefore, targeting each of the above steps such as migration and angiogenesis should be effective therapy for tumor metastasis.

Phosphatidylinositol 3-kinases (PI3Ks) are a family of lipid kinases that phosphorylate phosphatidylinositol 4,5-bisphosphate (PIP2) at the 3-hydroxyl group of the inositol ring to generate phosphatidylinositol 3,4,5-trisphosphate (PIP3) [2], which plays fundamental roles in cellular responses such as proliferation, survival as well as migration [3,4]. Frequent mutations found in the PIK3CA gene which encodes PI3Kα in human tumors suggest that PI3K is a potential target for cancer therapy [5]. Development of PI3K inhibitors has therefore attracted high attention from pharmaceutical industries [4,6]. Currently, over 20 PI3K inhibitors are under clinical evaluation. Among them, ZSTK474 is a novel PI3K inhibitor that we identified by use of JFCR39 drug discovery system [7–9]. ZSTK474 inhibits the four class I PI3K isoforms with high specificity over 141 protein kinases including mTOR and DNA-PK [10–12]. Preclinical study showed promising *in vitro* and *in vivo* antitumor efficacy on various solid tumors [8,13]. Besides antitumor activity, ZSTK474 also exhibited anti-inflammatory effect [14].

G1 cell cycle arrest and apoptosis induction have been well reported as antitumor mechanisms of the PI3K inhibitors such as ZSTK474 [8,15]. We previously reported that ZSTK474 exhibited antiangiogenic activity on vascular endothelial cells [13], suggesting the potential antimetastatic efficacy. However, antimetastatic activity of PI3K inhibitors has rarely been reported.

We recently investigated the antimetastatic activities of ZSTK474 by use of a hormone-refractory prostate cancer cell line PC3. In this paper, the inhibitory effects on migration, invasion and adhesion of PC3 cells are reported. Moreover, the activities on the expressions of HIF-1α and VEGF which play important roles in angiogenesis are described as well.

## 2. Results and Discussion

### 2.1. ZSTK474 Inhibited PC3 Proliferation

**Figure 1:**
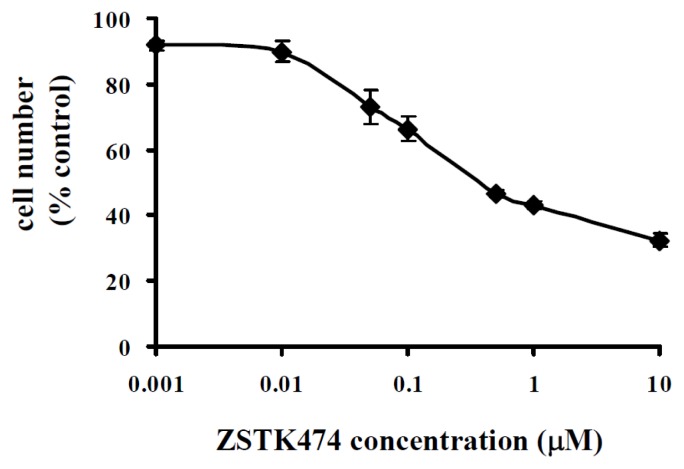
ZSTK474 inhibits proliferation of prostrate cancer PC3 cells. PC3 cells were incubated with various concentrations of ZSTK474 in 96-well plate at 37 °C. Two days later, WST-8 solution was added and the cells were further incubated for 3 h followed by measurement of the absorbance (450 nm) with microplate spectrophotometer. The number of remaining cells after treatment was calculated using the following formula: Cell number (% control) = 100 × (absorbance of a given sample − absorbance of Blank well)/(absorbance of Control well − absorbance of Blank well), where the Blank well contained medium only and the Control well contained cells without ZSTK474. Data are mean ± SD (*n* = 3), and are representative of three independent experiments.

We first examined the antiproliferative activity of ZSTK474 on PC3 cells by use of WST-8 assay as described by us previously [13]. After treatment with various concentrations of ZSTK474 for 48 h, the remaining cell number was measured. As shown in Figure 1, ZSTK474 dose-dependently inhibited the proliferation of PC3 cells. The IC50 (the concentration that causes 50% inhibition of the growth of cells) value was calculated to be 0.12 μM by use of GraphPad Prism 4 [16].

### 2.2. ZSTK474 Blocked PC3 Migration

**Figure 2:**
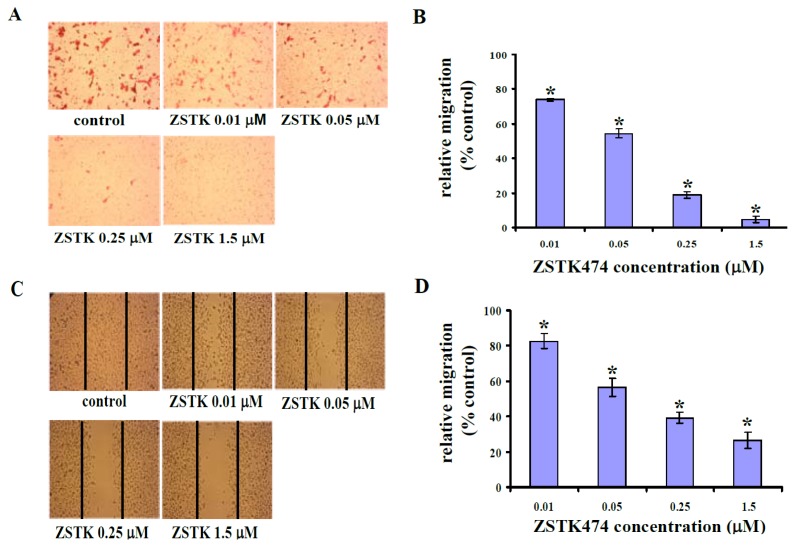
ZSTK474 inhibits migration of PC3 cells. (**A**) Representative images depicting the effect of treatment with the indicated concentrations of ZSTK474 on PC3 cell migration through the Transwell chamber membrane; (**B**) Percentages of PC3 cells migrated following treatment with ZSTK474 relative to those of the control cells (DMSO-treated); *: *p* < 0.01, compared with control. Data are mean ± SD (*n* = 3), and are representative of three independent experiments; (**C**) Representative images depicting the healing ability of PC3 cells with or without ZSTK474 treatment after wounded by a 0.2 mL pipette tip; (**D**) Percentages of PC3 cells migrated to the wound area following treatment with ZSTK474 relative to those of the control cells (DMSO-treated); *: *p* < 0.01, compared with control. Data are mean ± SD (*n* = 3), and are representative of three independent experiments.

To investigate the *in vitro* antimetastatic activity of ZSTK474, we first examined the effect on cell migration of PC3 cells using Transwell migration assay. Figure 2A shows the representative photographs of eosin-stained PC3 cells that migrated to the bottom face of the membrane. Thus, 0.01, 0.05, 0.25, and 1.5 μM of ZSTK474 treatment for 18 h inhibited the migration of PC3 cells by 26.2%, 45.6%, 70.0% and 95.3% (Figure 2B), respectively, suggesting that ZSTK474 inhibited PC3 cell migration in a dose-dependent manner.

To confirm the inhibitory activity on migration ability of PC3 cells, wound healing assay was used to determine the cell number that migrated to the wound area after treatment with ZSTK474. Figure 2C and Figure 2D indicated similar results to those obtained in the Transwell migration assay. Thus, ZSTK474 inhibited migration of PC3 cells in a dose-dependent manner.

### 2.3. ZSTK474 Reduced PC3 Invasion

**Figure 3:**
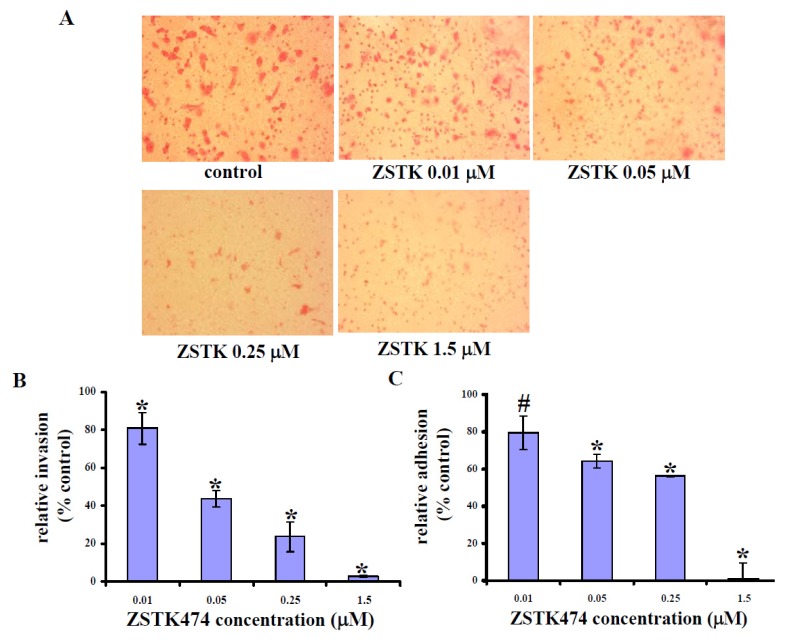
ZSTK474 inhibits invasion and adhesion of PC3 cells. (**A**) Representative photographs depicting the effect of treatment with the indicated concentrations of ZSTK474 on PC3 cell invasive ability through the Matrigel-coated Invasion chamber membrane; (**B**) Percentages of PC3 cells invaded through the Matrigel-coated Invasion chamber membrane following ZSTK474 treatment relative to those of the control cells (DMSO-treated); *: *p* < 0.01, compared with control. Data are mean ± SD (*n* = 3), and are representative of three independent experiments; (**C**) Percentages of PC3 cells adherent to fibronectin-coated plate well following ZSTK474 treatment relative to those of the control cells (DMSO-treated). #: *p* < 0.05; *: *p* < 0.01, compared with control. Data are mean ± SD (*n* = 3), and are representative of three independent experiments.

Cell invasion, including events like cell attachment, degradation of extracellular matrix (ECM) and migration, constitutes a key process for metastasis. We therefore examined the effect of ZSTK474 on PC3 cell invasive ability by use of Invasion Transwell chamber, of which the insert membrane was coated by Matrigel. As shown in Figure 3A, PC3 cells that invaded through the membrane decreased after treatment with ZSTK474 for 18 h. Thus, 0.01, 0.05, 0.25, and 1.5 μM of ZSTK474 inhibited the invasive ability of PC3 cells by 19.0%, 56.2%, 76.4% and 97.1% (Figure 3B), respectively, suggesting that ZSTK474 treatment inhibited PC3 cell invasion dose-dependently.

### 2.4. ZSTK474 Inhibited PC3 Adhesion

As a critical event, cell adhesion to ECM as well as adjacent cells is required for invasion and metastasis [17]. We therefore determined the effect of ZSTK474 on PC3 cell adhesive ability. The number of cells adherent to fibronectin-coated 96-well-plate was measured. In addition, the inhibitory activity of ZSTK474 against cell adhesion was correspondingly calculated. As a result, ZSTK474 treatment for 24 h reduced the number of cells adherent to fibronectin-coated wells dose-dependently. In particular, after treatment with 1.5 μM of ZSTK474, PC3 cells almost lost their adhesive ability completely (Figure 3C).

To summarize the above results, ZSTK474 exhibited inhibitory effects on proliferation, migration, invasion and adhesion of PC3 cells. By comparison of the concentrations of ZSTK474 showing in vitro antimetastatic effects and those for proliferation inhibition, and the times (18 h or 24 h *vs.* 48 h) used for antimetastatic assays and proliferation assay, we conclude that the antimetastatic effects of ZSTK474 are not attributed to the growth inhibitory activity,

Besides PC3 cells, *in vitro* antimetastatic effects of ZSTK474 on hormone-resistant prostate cancer cell DU145 were also investigated, and the result showed that ZSTK474 inhibited migration, invasion and adhesion of DU145 cells as well (data not shown).

### 2.5. ZSTK474 Inhibited Phosphorylation of Girdin in PC3 Cells

**Figure 4:**
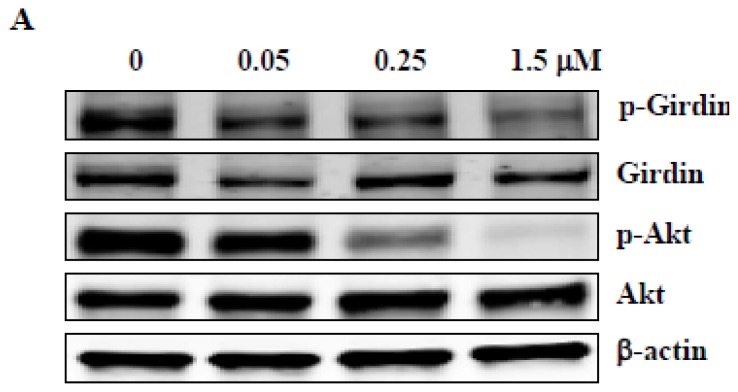

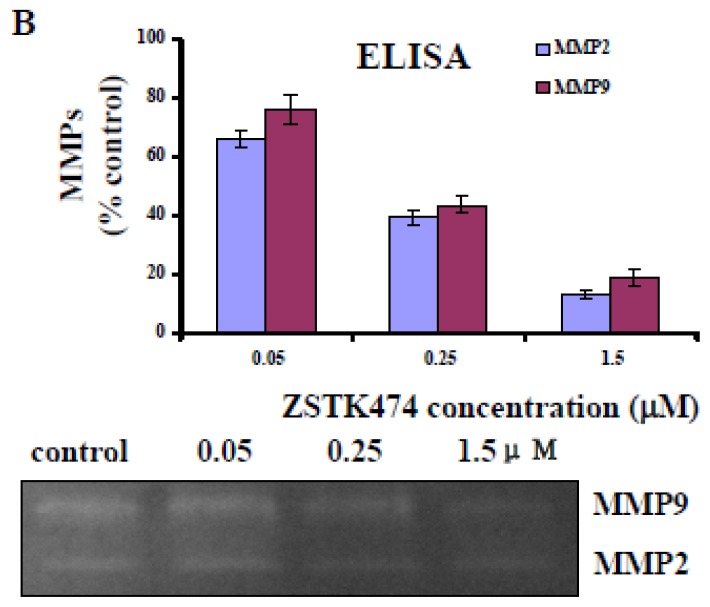
ZSTK474 inhibits phosphorylation of Girdin and blocks MMP secretion in PC3 cells. (**A**) ZSTK474 inhibits phosphorylation of Akt and its substrate Girdin in PC3 cells. The expression levels of p-Girdin, total Girdin, p-Akt, total Akt and β-actin in the cell lysates prepared from the DMSO-treated and ZSTK474-treated PC3 cells were determined by western blot analysis. β-actin expression was determined for a quantitative control of protein amount; (**B**) MMP2 and MMP9 secretion into the medium following treatment of PC3 cells with the indicated concentrations of ZSTK474. Data are mean ± SD (*n* = 3), and are representative of two independent experiments (upper panel); Reduced proteolytic activities of MMP2 and MMP9 shown by Gelatin zymography assay (lower panel).

Girdin, an actin-binding protein, was reported to mediate cell migration and invasion as a substrate of Akt [18,19]. To investigate the mechanism of ZSTK474 for migration inhibition, we examined the effect on phosphorylation of Girdin as well as Akt. As shown in Figure 4A, treatment with ZSTK474 for 24 h reduced the expression of p-Girdin as well as p-Akt with similar concentrations to those for migration and invasion inhibition. Densitometric analysis indicated treatment with 0.05, 0.25 and 1.5 μM ZSTK474 respectively reduced the expression to 81.9%, 73.2% and 60.7% for p-Girdin, and to 87.7%, 44.5% and 11.1% for p-Akt. Such results suggest that inhibition of the phosphorylation of Akt and the substrate Girdin might be involved in blockade of migration and invasion of PC3 cells.

### 2.6. ZSTK474 Inhibited the Secretion of MMP2 and MMP9 by PC3 Cells

Degradation of ECM surrounding primary tumors is necessary for tumor cell invasion. Matrix metalloproteinase (MMP) is a class of zinc-dependent proteolytic enzymes that are responsible for the degradation of ECM proteins [20]. To investigate the mechanism of ZSTK474 for inhibition against tumor cell invasion, we determined the effect on the secretion of MMP2 and MMP9 by PC3 cells. As shown in Figure 4B (upper panel), ZSTK474 treatment for 24 h decreased the secretion of MMP2 and MMP9 significantly even at a low concentration of 0.05 μM, with the inhibitory activity shown to be dose-dependent.

We also determined the effect of ZSTK474 on the proteolytic activities of MMP2 and MMP9 by gelatin zymography assay. Figure 4B (lower panel) indicated the activities of MMP2 and MMP9 in PC3 cell media after treatment with various concentrations of ZSTK474: ZSTK474 dose-dependently inhibited the proteolytic activities of MMP2 and MMP9.

### 2.7. ZSTK474 Inhibited the Expression of HIF-1α and VEGF in PC3 Cancer Cells

Angiogenesis, the process of generating new blood vessels from a primitive vascular network, is required for tumor metastasis [21]. Hypoxia inducible factor-1 (HIF-1) is a protein transcription factor composed of HIF-1α and HIF-1β subunit. While HIF-1β is constitutively expressed, HIF-1α is highly regulated which determines the activity of HIF-1. Besides hypoxia, HIF-1α can be activated by signals from pathways such as PI3K/Akt [22]. After activation, HIF-1α translocates into the nucleus and heterodimerizes with HIF-1β, therefore transcripts VEGF [23,24], which is well known to play a key role in angiogenesis [25,26].

**Figure 5:**
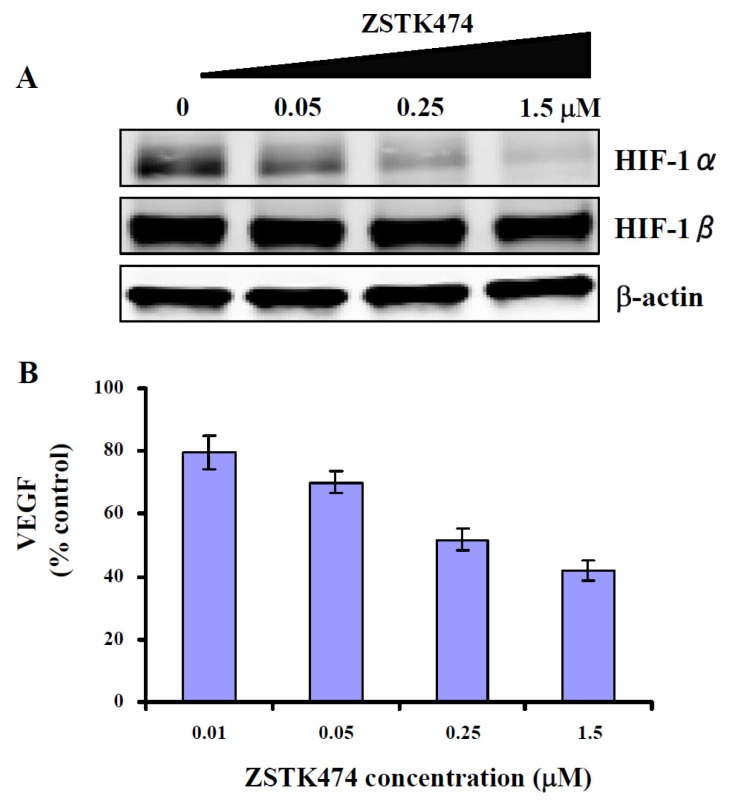
Potential antiangiogenic activity of ZSTK474 on PC3 cancer. (**A**) ZSTK474 inhibits the expression of hypoxia inducible factor-1 (HIF-1)α in PC3 cells. The expression levels of HIF-1α, HIF-1β and β-actin in the cell lysates prepared from the DMSO-treated and ZSTK474-treated PC3 cells were determined by western blot analysis. β-actin expression was determined for a quantitative control of protein amount; (**B**) VEGF secretion into the medium following treatment of PC3 cells with the indicated concentrations of ZSTK474. Data are mean ± SD (*n* = 3), and are representative of three independent experiments.

We previously reported that ZSTK474 inhibited HIF-1α expression and VEGF secretion in human renal cancer RXF-631 cells [13]. To demonstrate that ZSTK474 also acts on the angiogenesis caused by PC3 cells, we determined the expression of HIF-1α and secretion of VEGF in PC3 cells after ZSTK474 treatment for 24 h. Figure 5A showed the expression of HIF-1α and HIF-1β with or without treatment by ZSTK474. Treatment with 0.05, 0.25 and 1.5 μM of ZSTK474 dose-dependently reduced the expression of HIF-1α as 76.6%, 43.2% and 21.4%. As expected, expression of HIF-1β was not affected. Moreover, the secretion of VEGF which is the downstream effector of HIF-1α, was reduced by ZSTK474 dose-dependently (Figure 5B). The results suggest that ZSTK474 had the potential antiangiogenic activity on PC3 cancer, via blocking the HIF-1α expression and VEGF secretion in the PC3 cancer cells.

### 2.8. ZSTK474-Reduced VEGF Inhibited Tube Formation by HUVEC Cells

**Figure 6:**
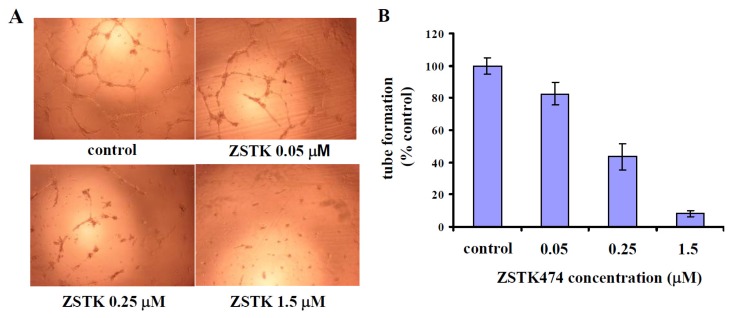
ZSTK474-reduced vascular endothelial growth factor (VEGF) inhibits tube formation by human umbilical vein endothelial cells (HUVECs). (**A**) Representative images depicting the tube formation by HUVEC following treatment with the conditioned media collected from PC3 cells after treatment with indicated concentrations of ZSTK474; (**B**) Percentage of the tube formation following treatment with the various conditioned media. Data are mean ± SD (*n* = 3), and are representative of three independent experiments.

To demonstrate that ZSTK474-reduced VEGF indeed affects angiogenesis, the conditioned media of PC3 cells after treatment with various concentrations of ZSTK474 were used to treat HUVEC cells. And the effects on capillary-like tube formation by HUVEC cells were analyzed. As indicated in Figure 6, media of the PC3 cells treated with various concentrations of ZSTK474 inhibited tube formation by HUVEC cells in a dose-dependent manner, compared with medium of control (PC3 cells treated with DMSO), demonstrating that ZSTK474 could exhibit the antiangiogenic effect via reducing VEGF secretion by cancer cells.

We previously reported the antiangiogenic activity of ZSTK474 by showing its inhibitory effect on the proliferation, migration and tube formation of human umbilical vein endothelial cells (HUVECs) [13]. Therefore, we conclude that ZSTK474 might inhibit PC3 tumor metastasis via two mechanisms: inhibition of tumor cell migration, invasion and adhesion; antiangiogenesis via blockade of VEGF secretion by PC3 cells and action on vascular endothelial cells. The dual mechanisms would contribute to the antimetastatic activity of ZSTK474 *in vivo*.

Angiogenesis inhibitors for tumor metastasis therapy raised significant interests among researchers [21,27]. Since the approval of Avastin by FDA of USA in 2004, dozens of angiogenesis inhibitors have been either approved or in clinical trials for the treatment of cancer. However, tumor metastasis has not yet been completely controlled by the angiogenesis inhibitors. This question is whether a pure angiogenesis inhibitor could produce satisfying antitumor efficacy for patients with tumor metastasis. We hypothesized that an antitumor agent targeting both tumor cell migration and angiogenesis might have better efficacy. ZSTK474 has the potential to inhibit both tumor cell migration and angiogenesis for PC3 cancer, is expected to become a promising drug candidate for therapy of prostate cancer with metastasis.

## 3. Experimental Section

### 3.1. Reagents

ZSTK474 was purchased from Selleck (London, ON, Canada). WST-8 assay kit was purchased from Kishida Chemicals (Osaka, Japan); anti-phospho-Akt (Ser473) and anti-Akt antibodies were from Cell Signaling Technology (Danvers, MA, USA); anti-phospho-Girdin (Ser1416) and anti-Girdin antibodies were from Immuno-Biological Laboratories Co., Ltd (Japan); anti-human HIF-1α and anti-human HIF-1β antibodies were from BD Biosciences (San Jose, CA, USA); anti-β-actin antibody was from Sigma-Aldrich (St. Louis, MO, USA). Quantikine^®^ human VEGF kit (Minneapolis, MN, USA) was purchased from R&D Systems (Minneapolis, MN, USA); human MMP2 ELISA kit (Cambridge, UK) was from Abcam (Cambridge, UK); human MMP9 ELISA kit was from Invitrogen (Now a part of Life Technologies, Carlsbad, CA, USA); matrigel was purchased from BD Biosciences (San Jose, CA, USA).

### 3.2. Cell Culture

Human prostate cancer PC3 cell line was obtained from American Type Culture Collection (ATCC, Manassas, VA, USA) and cultured in RPMI 1640 medium supplemented with 5% fetal bovine serum and kanamycin (100 U/mL) at 37 °C in a humidified atmosphere containing 5% CO_2_.

### 3.3. Cell Growth Inhibition Assay

Cell viability was determined using the WST-8 assay kit (Kishida Chemicals, Osaka, Japan), as described by us previously [13], with a little modification. One hundred μL of cells (6 × 10^4^ cells/mL) was seeded in 96-well plate and incubated at 37 °C. Twenty four hours later, 0.5 μL of various stock solutions of ZSTK474 was added. After further incubation for 48 h, 10 μL of WST-8 was added to each well and the cells were further incubated at 37 °C. Three hours later, the absorbances at 450 nm were measured with a microplate spectrophotometer (Bio Rad, Hercules, CA, USA). The number of viable cells remaining after the treatment was calculated using the following formula: Cell number (% control) = 100 × (absorbance of a given sample − absorbance of Blank well)/(absorbance of Control well − absorbance of Blank well), where the Blank well contained medium but no cells and the Control well contained cells but no ZSTK474. The IC_50_ value was calculated by fitting the data points to a logistic curve using the GraphPad Prism 4 software [16].

### 3.4. Transwell Migration Assay

The effect of ZSTK474 on cell migration *in vitro* was investigated by using a Transwell Boyden Chamber (Corning, MA, USA) containing a polycarbonate membrane with pore size of 8 μm as described by us previously [13]. Briefly, 0.1 mL of PC3 cell suspension (5 × 10^5^ cells/mL) in RPMI medium with various concentrations of ZSTK474 was placed to the upper compartment. The lower compartment contained 0.65 mL of RPMI supplemented with 10% FBS and the same concentration of ZSTK474 as that in the upper compartment. After incubation at 37 °C for 18 h, cells that migrated to the bottom face of the membrane were fixed with 90% ethanol, stained with 0.5% eosin, observed under the Olympus CKX41 microscope and photographed. The number of migrated cells was counted, and the percentage of PC3 cells migrated after the ZSTK474 treatment relative to that after the DMSO (vehicle solvent) treatment (control) was calculated. Student’s *t*-test was used to examine the difference of control and each treatment group. Representative data from three independent experiments, each carried out in triplicate, were used for plotting.

### 3.5. Wound Healing Assay

Wound healing assay was carried out as described by us previously [28] with a little modification. PC3 cells (1 × 10^6^ cells/mL) were allowed to grow into full confluence in 6-well plate. Then the monolayer cells were scratched with a 0.2 mL pipette tip. Fresh RPMI media were added with DMSO or with various concentrations of ZSTK474. After further incubation for 24 h, the migration was observed under the Olympus CKX41 microscope (Tokyo, Japan) and photographed. For quantification, the migrated cells were counted manually, and expressed as the percentages by comparison with the control (treated with DMSO). Student’s *t*-test was used to examine the difference of control and each treatment group. Three independent experiments were carried out.

### 3.6. Transwell Invasion Assay

Transwell invasion assay was used to investigate the effect of ZSTK474 on invasive ability of PC3 cells. The transwell chamber was pretreated with Matrigel (BD Biosciences, San Jose, MA, USA) (1:5 diluted in RPMI-1640), dried in room temperature for 2 h. 0.1 mL of PC3 cell suspension (1 × 10^6^ cells/mL) in RPMI medium with various concentrations of ZSTK474 was placed to the upper compartment. Other procedures and data analysis are the same as those described in the Transwell migration assay.

### 3.7. Cell Adhesion Assay

Cell adhesive ability was determined as reported [29] with a little modification. Briefly, 96 well plates were coated with 20 μg/mL of fibronectin or 1% BSA (bovine serum albumin, negative control) and incubated overnight at 37 °C. The coated wells were washed twice with PBS and dried in a clean bench. Suspensions of PC3 cells pretreated with various concentrations of ZSTK474 for 24 h were seeded into the coated wells and incubated for 2 h. The wells were further washed twice with PBS to remove the unattached cells. The adherent cells were then fixed with 90% ethanol and stained with crystal violet overnight. The absorbance at 570 nm was measured using a microplate reader (Bio Rad, Hercules, CA, USA). The signal of PC3 cells after the ZSTK474 treatment relative to that after the DMSO treatment (control) was calculated as relative adhesion. Student’s *t*-test was used to examine the difference of control and each treatment group. Three independent experiments were carried out.

### 3.8. Western Blot Analysis

Western blot analysis was carried out as described by us previously [13]. Cell lysates from ZSTK474-treated and control (DMSO-treated) cells were prepared. Proteins in the cell lysates were separated by sodium dodecyl sulfate-polyacrylamide gel electrophoresis (SDS-PAGE), and then transferred onto Immobilon-FL membranes (Millipore, Billerica, MA, USA). After being blocked, the membranes were incubated with the first antibodies, and then the respective secondary antibodies. Signals from the bound labeled-antibodies were detected using Odyssey Infrared Imaging System (LI-COR Biosciences, Lincoln, NE, USA). To confirm equal protein loading, the bound antibodies were stripped off from the blots by the stripping buffer (25 mM glycine pH 2.0 and 2% SDS) and then re-probed with anti-β-actin antibody. For quantification of the expression of p-Akt, p-Girdin and HIF-1α, densitometric analysis was carried out by use of Image J software [30].

### 3.9. Enzyme-Linked Immunosorbent Assay (ELISA)

To investigate the effect of ZSTK474 on VEGF secretion by PC3 cells, ELISA was performed as described by us previously [13]. Sub-confluent PC3 cells in a 6-well plate were treated with various concentrations of ZSTK474 for 24 h. The culture medium from each well was collected and centrifuged. The supernatants were stored at −20 °C to be available for ELISA. Quantikine^®^ human VEGF kit from R&D Systems (Minneapolis, MN, USA) was used to quantify the VEGF in the supernatants. The relative amount in the ZSTK474-treated cells was expressed as the percentage of amount in the DMSO-treated control cells.

For determination of the secretion of MMP2 and MMP9, human MMP2 ELISA kit from Abcam (Cambridge, UK) and human MMP9 ELISA kit from Invitrogen (Carlsbad, CA, USA) were used respectively. Preparation of the medium supernatant was carried out the same as that described above. The resulting supernatants were concentrated by Amicon Ultra-4 Centrifugal Filter (Millipore, Billerica, MA, USA) to be available for MMP2 and MMP9 ELISA. The relative MMP amounts in the ZSTK474-treated cells were expressed as the percentages of those in the DMSO-treated control cells.

### 3.10. Gelatin Zymography Assay

The proteolytic activities of MMP2 and MMP9 in media of PC3 cells were determined by gelatin zymography assay as reported previously [31] with a little modification. Briefly, the media of PC3 cells after treatment with various concentrations of ZSTK474 were collected and concentrated by Amicon Ultra-4. The resulting samples were mixed with SDS loading buffer, subjected to 0.1% gelatin and 8% SDS-PAGE. Afterwards, the gel was washed with 2.5% Triton X-100 and then incubated for 24 h at 37 °C in reaction buffer (50 mM Tris-HCl, pH 7.5, 10 mM CaCl_2_, 0.01% NaN_3_). Then the gel was stained with Coomassie Brilliant Blue R-250 (Merck, Darmstadt, Germany).

### 3.11. Tube Formation Assay

The effect of ZSTK474-reduced VEGF on tube formation *in vitro* was determined as described by us previously [13] after a little modification. Briefly, pre-chilled 96-well plate was coated with matrigel (BD Biosciences, San Jose, CA). 150 μL of HUVECs (2 × 10^5^ cells/mL) in medium of endothelial cell growth medium-2 (EGM2 BulletKit, Clonetics, Basel, Switzerland) mixed (1:1) with conditioned medium from ZSTK474-treated PC3 cells as indicated in *3.9* was seeded in each well of the coated plate. The cells were then incubated for 18 h at 37 °C. The capillary-like tubes formed were visualized under the Olympus CKX41 microscope. Representative network of tube structures formed in each well was photographed. For quantification, the total length of the tubes was measured using the MetaMorph 6.3 software [32], and from the data, percentage of tubes formed in each ZSTK474-treated well relative to the control well (DMSO-treated cells) was calculated. Representative data from three independent experiments were used for analysis.

## 4. Conclusions

Our present study demonstrated that ZSTK474 inhibited migration, invasion and adhesion of PC3 cells. The effects might be at least partially attributed to the inhibition against phosphorylation of Akt and its substrate Girdin through PI3K/Akt pathway. Moreover, ZSTK474 exhibited potential antiangiogenic effects on PC3 cells via inhibiting the expression of HIF-1α and the secretion of VEGF. In summary, ZSTK474 might inhibit PC3 tumor metastasis via two mechanisms: inhibition of tumor cell migration, invasion and adhesion; antiangiogenesis via blockade of VEGF secretion by PC3 cells.
